# Indomethacin Induces Spermidine/Spermine-N^1^-Acetyltransferase-1 via the Nucleolin-CDK1 Axis and Synergizes with the Polyamine Oxidase Inhibitor Methoctramine in Lung Cancer Cells

**DOI:** 10.3390/biom13091383

**Published:** 2023-09-12

**Authors:** Neudo Buelvas, Isidora Ugarte-Vio, Laura Asencio-Leal, Matías Muñoz-Uribe, Antonia Martin-Martin, Alejandro Rojas-Fernández, José A. Jara, Julio C. Tapia, María Elena Arias, Rodrigo A. López-Muñoz

**Affiliations:** 1 Instituto de Farmacología y Morfofisiología, Facultad de Ciencias Veterinarias, Universidad Austral de Chile, Valdivia P.O. Box 5110566, Chile; 2 Instituto de Medicina, Facultad de Medicina, Universidad Austral de Chile, Valdivia P.O. Box 5110566, Chile; 3 Instituto de Investigaciones en Ciencias Odontológicas (ICOD), Facultad de Odontología, Universidad de Chile, Santiago P.O. Box 8380544, Chile; 4 Programa de Biología Celular y Molecular, Instituto de Ciencias Biomédicas, Facultad de Medicina, Universidad de Chile, Santiago P.O. Box 8380453, Chile; 5 Departamento de Producción Agropecuaria, Universidad de La Frontera, Temuco P.O. Box 4811230, Chile

**Keywords:** polyamines, cancer, indomethacin, non-steroidal anti-inflammatory drugs

## Abstract

Indomethacin is a non-selective NSAID used against pain and inflammation. Although cyclooxygenase (COX) inhibition is considered indomethacin’s primary action mechanism, COX-independent ways are associated with beneficial effects in cancer. In colon cancer cells, the activation of the peroxisome proliferator-activated receptor-γ (PPAR-γ) is related to the increase in spermidine/spermine-N^1^-acetyltransferase-1 (SSAT-1), a key enzyme for polyamine degradation, and related to cell cycle arrest. Indomethacin increases the SSAT-1 levels in lung cancer cells; however, the mechanism relying on the SSAT-1 increase is unclear. Thus, we asked for the influence of the PPAR-γ on the SSAT-1 expression in two lung cancer cell lines: H1299 and A549. We found that the inhibition of PPAR-γ with GW9662 did not revert the increase in SSAT-1 induced by indomethacin. Because the mRNA of SSAT-1 suffers a pre-translation retention step by nucleolin, a nucleolar protein, we explored the relationship between indomethacin and the upstream translation regulators of SSAT-1. We found that indomethacin decreases the nucleolin levels and the cyclin-dependent kinase 1 (CDK1) levels, which phosphorylates nucleolin in mitosis. Overexpression of nucleolin partially reverts the effect of indomethacin over cell viability and SSAT-1 levels. On the other hand, Casein Kinase, known for phosphorylating nucleolin during interphase, is not modified by indomethacin. SSAT-1 exerts its antiproliferative effect by acetylating polyamines, a process reverted by the polyamine oxidase (PAOX). Recently, methoctramine was described as the most specific inhibitor of PAOX. Thus, we asked if methoctramine could increase the effect of indomethacin. We found that, when combined, indomethacin and methoctramine have a synergistic effect against NSCLC cells in vitro. These results suggest that indomethacin increases the SSAT-1 levels by reducing the CDK1-nucleolin regulatory axis, and the PAOX inhibition with methoctramine could improve the antiproliferative effect of indomethacin.

## 1. Introduction

Non-steroidal anti-inflammatory drugs (NSAIDs) constitute a chemically diverse group of molecules utilized for pain and inflammation treatment. They exert their effects by inhibiting cyclooxygenases (COX-1 and COX-2), which are pivotal enzymes involved in the synthesis of prostaglandin E_2_ (PGE_2_), thromboxane A_2_ (TXA_2_), and prostacyclin (PGI_2_) [1]. Apart from their anti-inflammatory properties, NSAIDs have demonstrated antitumor and chemopreventive effects in gastrointestinal tumors [2,3]. Furthermore, they have been associated with a negative correlation with distant metastases in prostate and breast cancer patients [4], and perioperative NSAID use has shown a significant increase in disease-free survival among breast and ovarian cancer patients [5]. Additionally, certain studies suggest a moderate reduction in the risk of lung cancer associated with NSAIDs, particularly acetylsalicylic acid [6,7,8].

Some of these antitumor effects are explained by the inhibition of the COX enzymes because prostaglandins play pivotal roles in the development and growth of digestive, breast, skin and lung cancer, among others [9]. However, the activation of nuclear factor kappa-light-chain-enhancer of activated B cells (NF-κB), the generation of specialized pro-resolving mediators (SPMs), and the activation of the peroxisome proliferator-activated receptor-γ (PPAR-γ), among others, have been proposed as alternatives modes of actions beyond COX blockade [10,11].

Indomethacin, a non-selective NSAID with the ability to inhibit both COX-1 and COX-2, is widely used for the reduction of moderate pain and inflammation. Additionally, its COX-independent mechanisms have been associated with beneficial effects in cancer. For instance, indomethacin sensitizes drug-resistant tumor cells by reducing the overexpression of the multidrug resistance protein 1 (MRP1) [12,13]. It also induces mitochondrial dysfunction and apoptosis in vitro [14]. Moreover, indomethacin acts as an agonist of PPAR-γ, exhibiting similar activity to other NSAIDs like S-naproxen but with greater potency compared to ibuprofen. This suggests that not all NSAIDs have equal capability to activate this receptor [15].

Indomethacin and other NSAIDs have been found to induce the expression of an enzyme called spermidine/spermine N1-acetyltransferase (SSAT-1) [16,17,18,19]. SSAT-1 is encoded by the SAT1 gene and is responsible for acetylating polyamines, facilitating their export from mammalian cells [20]. Polyamines, including putrescine, spermidine, and spermine, are essential for various cellular processes, such as cell cycle regulation, redox balance, immune modulation, and cell growth [21]. Elevated levels of polyamines are observed in several tumor types, including lung cancer [22,23]. The increase in SSAT-1 leads to a decrease in intracellular polyamine levels and subsequently induces cell cycle arrest [24]. However, mammalian cells can recover polyamines after acetylation through the action of polyamine oxidase (PAOX), which converts acetyl-spermine to spermidine and acetyl-spermidine to putrescine [25]. Methoctramine has recently been identified as a highly specific inhibitor of PAOX, preventing this recovery process and enhancing the accumulation of acetylated polyamines [26].

In colon cancer cells, certain NSAIDs, such as sulindac, induce apoptosis and suppress carcinogenesis by activating the SAT1 gene transcriptionally. Aspirin, at therapeutic concentrations, can also induce SSAT-1 mRNA through transcription initiation mechanisms. This induction leads to increased SSAT-1 levels and enzymatic activity [17]. These findings suggest that the upregulation of SSAT-1 by various NSAIDs could play a crucial role in chemopreventive strategies for colorectal cancer and potentially other tumor types [27].

Previously, we demonstrated that indomethacin increases SSAT-1 levels in non-small cell lung cancer (NSCLC) cells, altering polyamine metabolism and inducing cell death [19]. However, the specific mechanisms underlying indomethacin’s effects on polyamine metabolism are not fully understood. In this study, we present evidence suggesting that the increase in SSAT-1 by indomethacin is associated with post-transcriptional regulators of SSAT-1 mRNA, namely, nucleolin and Cyclin-dependent kinase 1 (CDK1 or Cell Division Cycle protein 2 homolog, CDC2). Additionally, we propose that the PAOX inhibitor methoctramine could enhance the effectiveness of indomethacin in NSCLC cells.

## 2. Materials and Methods

### 2.1. Cell Culture

The human NSCLC cell lines A549 (CCL-185™) and H1299 (CRL-5803™) were purchased from the American Type Culture Collection (ATCC, Manassas, VA, USA). Both cell ines used in this study harbor different genetic fingerprints. Whereas H1299 are null for the p53 protein and the wild type for KRAS, A549 cells are p53 wild type and carry the G12S mutation in the KRAS protein [28,29]. The cells were cultured in humidified air and 5% CO_2_ using RPMI 1640 culture media, supplemented with 10% fetal bovine serum (FBS) and antibiotics (penicillin 100 U/mL and streptomycin 100 μg/mL). Cells were used for ≤20 passages.

### 2.2. Drugs and Experimental Design

Indomethacin and GW9662 were purchased from Cayman Chemical (Ann Arbor, MI, USA), and methoctramine was purchased from SigmaAldrich (St. Louis, MO, USA). All of the drugs were dissolved in dimethyl sulfoxide (DMSO). DMSO was pumped with nitrogen for one minute before using it to avoid drug oxidation. Aliquots of drugs were stored at −80 °C until used and discarded after the thawing. For all experiments, the maximal concentration of DMSO in the culture medium was 0.5%, which did not influence cell viability. For immunoblotting, the cells were exposed to the drugs for 24 h, as previously described [19]. For the cell viability and combination studies, the cells were exposed to at least six concentrations of each drug for 72 h, as described in the results section.

### 2.3. Immunoblotting

We seeded H1299 and A549 cells in six-well plates (1 × 10^6^ cells per well) and allowed them to attach for 24 h. Then, we added indomethacin, GW9662, or a combination of them for 24 h. Cell lysates were prepared using PierceTM RIPA Buffer (Thermo Fisher Scientific, Rockford, IL, USA) containing 25 mM Tris–HCl (pH 7.6), 150 mM NaCl, 1% NP-40, 1% sodium deoxycholate, and 0.1% sodium dodecyl sulfate (SDS), supplemented with HaltTM Protease Inhibitor Cocktail (Thermo Fisher Scientific, Rockford, IL, USA). Samples were placed on ice for 10 min and centrifuged for 15 min at 15,000× *g*. Total protein concentration was determined using the Protein Assay Dye Reagent Concentrate (Bio-Rad Laboratories, Hercules, CA, USA). Subsequently, 80 μg of total protein were denaturated to 95 °C for 5 min and resolved using 15% SDS-polyacrylamide gel electrophoresis at 25 mA. Proteins were electrotransferred to a ProtranTM Pure Nitrocellulose Membrane 0.45 Micron (PerkinElmer, Waltham, MA, USA) at 450 mA for 1 h. The membrane was blocked using 5% non-fat milk in Tris-buffered saline containing 0.1% Tween 20 (TBST, pH 7.4) for 1 h at room temperature and washed with TBST. The membranes were incubated with the following primary antibodies: anti-SSAT-1 (Cat #61586, Cell Signaling Technologies, Danvers, MA, USA), anti-nucleolin (Cat #39-6400, Invitrogen, Waltman, MA, USA), anti-CDK1 (CAT #BSM-52026R, Thermofisher-Bioss Antibodies, Woburn, MA, USA), anti-CK2 alpha-2 (Cat #MA5-17062, Invitrogen, Waltman, MA, USA), anti-vinculin (Cat #sc-73614, Santa Cruz Biotechnology Inc., Dallas, TX, USA), or anti-β-actin (Cat #sc-47778, Santa Cruz Biotechnology Inc., Dallas, TX, USA). The antibodies were used at a dilution of 1:1000 for SSAT-1, nucleolin, CK2 and CDK1 antibodies, 1:10,000 for the vinculin antibody, or 1:2000 for the β-actin antibody in 5% non-fat milk in 0.1% TBST at 4 °C overnight. The membranes were washed with 0.1% TBST and incubated with a secondary anti-rabbit immunoglobulin G conjugated to horseradish peroxidase (IgG-HRP) (Cat #sc-2004 1:5000) or anti-mouse IgG-HRP (Cat #115-035-003, 1:5000) antibody in 5% milk in 0.1% TBST. Anti-rabbit IgG-HRP antibody was purchased from Santa Cruz Biotechnology (Dallas, TX, USA), and the anti-mouse IgG-HRP was purchased from Jackson ImmunoResearch Laboratories (West Grove, PA, USA). Following incubation, the membranes were washed with 0.1% TBST. Blots were imaged using an LI-COR Odyssey^®^ Fc immunoblot scanner (Lincoln, NE, USA). Immunoblot quantitation was performed using the Image Studio Lite Version 5.0 (LI-COR Biosciences, Lincoln, NE, USA). Uncropped scans for the immunoblots are shown in the Figure S1.

### 2.4. Immunofluorescence Imaging

We seeded 10,000 cells per well in black 96-well plates with a clear bottom. After 24 h, we exposed the cells to indomethacin (1 mM) or celecoxib (100 µM) for 24 h. Then, cells were washed three times with phosphate-buffered saline (PBS) and fixed with 4% of paraformaldehyde (PFA) for 30 min at room temperature. Cells were then washed twice with PBS and then permeabilized with 0.2% of Triton X-100 in PBS for 30 min. The plate was then blocked for 1 h with a FBS solution (5%) in PBS. Cells were then incubated with anti-SSAT-1 antibody (Cat #61586, Cell Signaling Technologies, Danvers, MA, USA) at a 1:1000 dilution for 45 min at 37 °C. The unbounded antibody was washed three times with PBS. An anti-rabbit linked to AlexaFluor-488 was used as the secondary antibody (Cat #A-11034, Invitrogen^®^, Thermo Fisher Scientific, Rockford, IL, USA) and was incubated for 35 min at 37 °C at a 1;10,000 dilution. Cells were then washed three times with PBS. After, we incubated the cells with DAPI for 10 min in darkness, and the plate was then washed three times with PBS, left with 100 µL of PBS, sealed and left at 4 °C until image capturing. Images were acquired with a Zeiss CellDiscoverer7 automated microscope (Carl Zeiss AG, Oberkochen, Germany). SSAT-1 was detected by using with wavelengths of 488 and 509 nm for excitation and emission, respectively, whereas DAPI was detected by using wavelengths of 353 and 465 nm for excitation and emission, respectively.

### 2.5. Cell Viability Measurement

NSCLC cells were seeded in 96 wells at a density of 2.5 × 10^3^ (for H1299 cells) or 5 × 10^3^ (for A549 cells) cells per well. After 24 h, cells were exposed to the drugs for 96 h. Subsequently, cell viability was evaluated through incubation with resazurin for 4 h. The production of resorufin (the fluorescent product of resazurin reduction by cells) was measured at 560 nm (excitation) and 590 nm (emission) in a Infinity200 Pro multimode reader (TECAN gmbH, Grödig, Austria).

### 2.6. Nucleolin Overexpression

We transiently overexpressed nucleolin in H1299 cells by transfecting them with a plasmid containing the nucleolin coding sequence, which was generously provided by Dr. Ronald T. Hay of the University of Dundee (Scotland). For transfection experiments, we used the NSCLC cell line H1299. H1299 cells were cultured in RPMI1640 medium supplemented with 10% fetal bovine serum and antibiotic-antimycotic (Penicillin/Streptomycin solution 100 U/mL). The transfection protocol suggested by the Lipofectamine^TM^ 3000 provider (Invitrogen, Waltham, MA, USA) was followed. Viable H1299 cells were seeded in 60-mm Petri dishes and allowed to adhere until reaching a confluence of 70–80%. Then, 5.5 µg of the nucleolin-CFP plasmid were administered. One day before transfection, 250,000 cells were plated in 3 mL of DMEM medium in 60-mm cell culture plates (Corning, Corning, NY, USA) to ensure a 70–80% cell confluence at the time of transfection. Transfection was then carried out in serum-free Opti-MEM medium (ThermoFisher Scientific, Waltham, MA, USA), for better compatibility with Lipofectamine^TM^ 3000. For each plate to be transfected, 5.5 μg of the nucleolin-CFP vector was diluted in 250 μL of serum-free Opti-MEM and mixed gently. Separately, 16.5 μL of Lipofectamine was diluted in 250 μL of Opti-MEM. The diluted DNA was then combined with the diluted Lipofectamine^TM^, mixed gently, and incubated for 15 min at room temperature. The culture medium was removed, and 500 μL of the diluted complexes were added to the plate and mixed gently by swirling. Subsequently, 2.5 mL of complete RPMI medium was added, and the cells were incubated at 37 °C in a 5% CO_2_ incubator for 24 h. Finally, the transfection efficiency was tested by evaluating nucleolin expression by Western blot at 24–72 h post-transfection.

### 2.7. Drug Combination Studies

Experiments of indomethacin combined with methoctramine were conducted to evaluate their synergistic effects, according to the Bliss models [30]. Viability was evaluated at 96 h through resazurin reduction, and data were analyzed using the COMBENEFIT software (Ver. 2.02) [31].

### 2.8. Statistical Analyses

For the western blotting studies, data were analyzed using the GraphPad Prism software (V 9.0, GraphPad Software, San Diego, CA, USA). One-way analysis of variance was performed to compare groups. Dunnett’s post-test was used to compare data with the control group, while Tukey’s post-test was used to perform comparisons between all experimental groups. The combination studies were evaluated using the *t*-test to compare differences between the theoretical model of Bliss and the obtained data. For all comparisons, a *p* < 0.05 denoted statistical significance.

## 3. Results

### 3.1. Indomethacin Increases the SSAT-1 Protein Levels, Independent of the Activation of PPAR-γ

**Figure 1:**
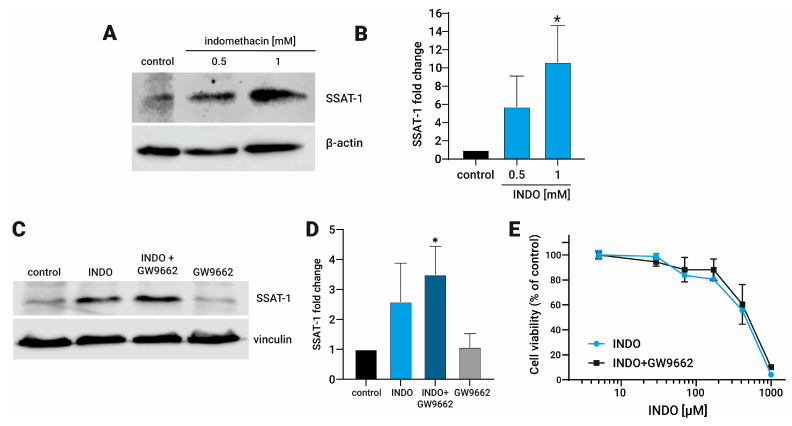
Effect of indomethacin on the spermidine/spermine-N^1^-acetyltransferase-1 (SSAT-1) levels and its relationship with the inhibition of the peroxisome proliferator-activated receptor-γ (PPAR-γ). H1299 were exposed to indomethacin 0.5 and 1 mM with or without the PPAR-γ inhibitor GW9662 (10 µM) for 24 h. The levels of SSAT-1 were evaluated by immunoblotting. (**A**) Representative blot of the SSAT-1 protein levels in H1299 cells exposed to indomethacin (0.5 and 1 mM). (**B**) Quantitation of the SSAT-1 levels in H1299 cells exposed to indomethacin (1 mM). (**C**) Representative blot of the SSAT-1 protein levels in H1299 cells exposed to indomethacin 1 mM and the PPAR-γ inhibitor GW9662 (10 µM). (**D**) Quantitation of the SSAT-1 levels in H1299 cells exposed to indomethacin (1 mM). (**E**) Cell viability of H1299 after 96 hrs. of exposition to indomethacin alone, or in presence of GW9662 (10 µM). Panel (**B**,**D**) show relative expression levels that were normalized using the control value from each individual replicate. *: *p* < 0.05, compared with control, calculated using one-way ANOVA and Dunnet’s post-test. Data summarize the results of three independent experiments. Original Images can be found in Figure S1.

We have previously reported that indomethacin increased the mRNA levels of SAT1 and the SSAT-1 protein levels in H1299 and A549 lung cancer cells [19]. Here, we confirm in a new set of experiments that indomethacin increases the SSAT-1 protein levels in 5.7 ± 3.37-fold at 0.5 mM (*p* = 0.173) and 10.6 ± 4.03-fold at 1 mM (*p* = 0.014) in H1299 cells (Figure 1A,B). As indomethacin can acts as an agonist of PPAR-γ [15], we seek whether an inhibitor, GW9662 (10 µM), may reverse its effect. As observed, this increase was not reversed by the PPAR-γ inhibitor GW9662 (INDO: 2.59 ± 1.28, *p* = 0.109; INDO + GW9662: 3.49 ± 0.94, *p* = 0.0154; GW9662: 1.08 ± 0.45, *p* = 0.999), suggesting that the PPAR-γ activation is not the unique factor that influences the increase in the SSAT-1 protein levels in H1299 cells (Figure 1C,D). Of note, GW9662 was also unable to revert the cytotoxic effect of indomethacin over the NSCLC cell line H1299 (Figure 1E).

### 3.2. The Increment in SSAT-1 Levels Correlates with a Decrease in Nucleolin in Lung Cancer Cells Exposed to Indomethacin

**Figure 2:**
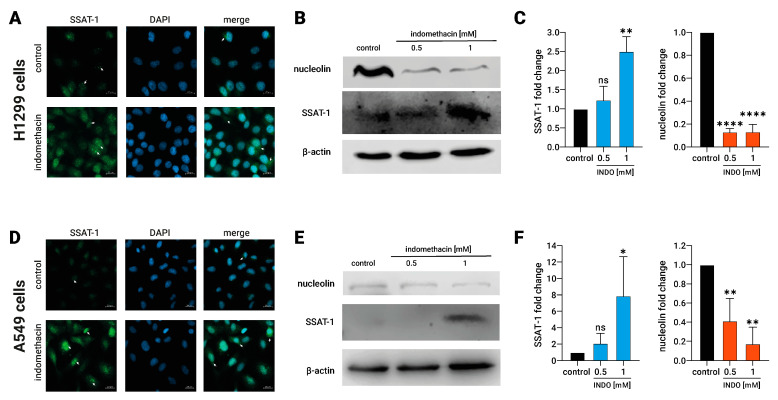
Effect of indomethacin on the spermidine/spermine-N^1^-acetyltransferase-1 (SSAT-1) and nucleolin levels in lung cancer cell lines. H1299 and A549 cells (non-small cell lung cancer) were exposed to indomethacin 0.5 and 1 mM for 24 h. (**A**) SSAT-1 detection by immunofluorescence microscopy in H1299 cells exposed to 0.5 mM indomethacin. DAPI was used for staining the nuclei. (**B**) Representative blot of the SSAT-1 and nucleolin protein levels in H1299 cells exposed to indomethacin. (**C**) Quantitation of the SSAT-1 and nucleolin levels in H1299 cells exposed to indomethacin. (**D**) SSAT-1 detection by immunofluorescence microscopy in A549 cells exposed to 0.5 mM indomethacin. DAPI was used for staining the nuclei. (**E**) Representative blot of the SSAT-1 and nucleolin protein levels in A549 cells exposed to indomethacin (**F**) Quantitation of the SSAT-1 and nucleolin levels in A549 cells exposed to indomethacin. For (**A**,**D**) panels, white arrows show nuclear spots of SSAT-1 (SSAT-1 images) and cytosolic expression of SSAT-1 (merge panels). Panel (**C**,**F**) show relative expression levels that were normalized using the control value from each individual replicate. *: *p* < 0.05; **: *p* < 0.01; ****: *p* < 0.0001, compared with control, calculated using one-way ANOVA and Dunnet’s post-test. Data summarize the results of three independent experiments. Original Images can be found in Figure S1.

It has been described that nucleolin exerts a pivotal regulation of the SSAT-1 translation by direct binding to the SSAT-1 mRNA. Consequently, a reduction of nucleolin by siRNA increases the SSAT-1 protein expression [32]. Therefore, we assessed if increased SSAT-1 after indomethacin exposure could be associated with a reduction of nucleolin levels. First, we evaluated the nucleolin and SSAT-1 levels in NSCLC cells treated with indomethacin. As expected, we confirmed the increased SSAT-1 levels in both H1299 and A549 cells by immunofluorescence microscopy (Figure 2A,D). Notably, cells without indomethacin showed low expression and nuclear localization of SSAT-1. After indomethacin 1 mM exposure for 24 h, nuclear spots of SSAT-1 become more intense, and SSAT-1 appears with great intensity in the cytoplasm.

In addition, H1299 and A549 cells were exposed to 1 mM indomethacin for 24 h, and then SSAT-1 and nucleolin levels were evaluated by immunoblotting. In H1299, indomethacin led to a significantly increases of the SSAT-1 protein levels at 1 mM (INDO 1 mM: 2.5 ± 0.4-fold over the control; *p* = 0.0016) and decreases in the nucleolin protein levels (INDO 1 mM: 0.13 ± 0.06, compared with control; *p* < 0.0001, Figure 2B,C). The same effect of 1 mM indomethacin was observed in A549 cells. Indomethacin also significantly increases the levels of SSAT-1 at 1 mM (INDO 1 mM: 7.9 ± 4.8-fold over the control; *p* = 0.0441), decreasing, at the same time, the levels of nucleolin (INDO 1 mM: 0.17 ± 0.17, compared with control; *p* = 0.0018, Figure 2E,F). This result suggests a novel nucleolin-dependent mechanism behind increased SSAT-1 after indomethacin exposure.

### 3.3. Indomethacin Decreases the CDK1 Protein Levels in Lung Cancer Cells

Nucleolin function is regulated by two protein kinases, namely, the casein kinase (CK2) and the cyclin-dependent kinase 1 (CDK1) [33], whereas CK2 phosphorylates nucleolin at six different residues of its N-terminal [34] and CDK1 phosphorylates nucleolin at the Thr-641/707 residues, increasing its stability during mitosis [35]. It has also been noted that 600 µM indomethacin significantly reduces the CDK1 levels in colon cancer cells [36].

**Figure 3:**
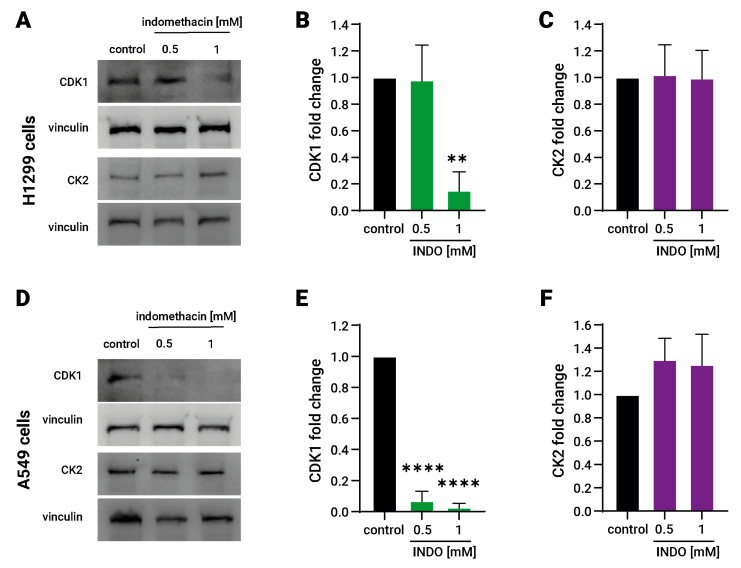
Effect of indomethacin on the Cdk1 and CK2 levels in lung cancer cell lines. H1299 and A549 cells (non-small cell lung cancer) were exposed to indomethacin 0.5 and 1 mM for 24 h. CDK1 and CK2 were detected by immunoblotting. (**A**) Representative blots of the CDK1 and CK2 protein levels in H1299 cells exposed to indomethacin. (**B**) Quantitation of the CDK1 levels in H1299 cells exposed to indomethacin. (**C**) Quantitation of the CK2 levels in H1299 cells exposed to indomethacin. (**D**) Representative blot of the SSAT-1-1 and nucleolin protein levels in A549 cells exposed to indomethacin. (**E**) Quantitation of the CDK1 levels in A549 cells exposed to indomethacin. (**F**) Quantitation of the CK2 levels in H1299 cells exposed to indomethacin. Panel (**B**,**C**,**E**,**F**) show relative expression levels that were normalized using the control value from each individual replicate. **: *p* < 0.01; ****: *p* < 0.0001, compared with control, calculated using one-way ANOVA and Dunnet’s post-test. Data summarize the results of three independent experiments. Original Images can be found in Figure S1.

To address whether indomethacin affects these upstream nucleolin regulators, we evaluated the protein levels of CK2 and CDK1 after exposure to indomethacin for 24 h by immunoblotting. CDK1 levels decreased significantly in both lung cancer cell types. In H1299, CDK1 levels decreased to approximately 15% at 1 mM indomethacin (CDK1 = 0.15 ± 0.14, *p* = 0.0017 compared with control). In A549 cells, 0.5 and 1 mM indomethacin reduced significantly the CDK1 levels approximately to 7% and 3%, respectively (INDO 0.5 = 0.069 ± 0.062 and INDO 1 mM = 0.028 ± 0.026, *p* < 0.0001, for both treatments compared with control). However, CK2 levels remained unchanged after indomethacin exposure in both H1299 and A549 cells (Figure 3A,C,D,F). Although CK2 remains unaltered, it seems that its activity is not important as an indomethacin mediator because inhibition of CK2 with CX-4945 did not revert the effect of indomethacin over SSAT-1 levels or the cell viability. Overall, these results suggest that CDK1 down-regulation by indomethacin in lung cancer cells leads to a decrease in nucleolin levels and ultimately increases SSAT-1.

### 3.4. Nucleolin Prevent the Increase in SSAT-1 and Partially Revert the Effect of Indomethacin

**Figure 4:**
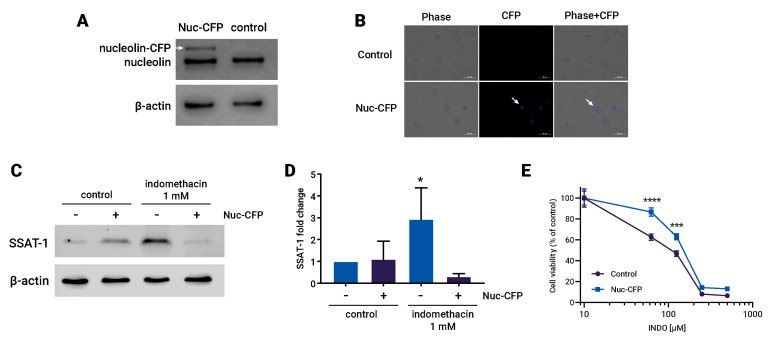
Overexpression of nucleolin reverses the effect of indomethacin over spermidine/spermine-N1-acetyltransferase-1 (SSAT-1) levels and cell viability. H1299 cells were transfected with a Nucleolin-CFP (Cyan Fluorescent Protein) expressing vector and exposed to indomethacin. (**A**) Immunoblot of nucleolin in H1299 cells transfected with nucleolin-CFP vector. White arrow shows the nucleolin protein that carry the CFP. (**B**) Fluorescence microscopy showing the CFP fluorescence in transfected H1299 cells. (**C**) Representative blot of the SSAT-1 protein levels in H1299 cells transfected with the nucleolin-CFP vector and exposed to indomethacin 1 mM for 24 h. (**D**) Quantitation of the SSAT-1 levels in H1299 cells ells transfected with the nucleolin-CFP vector and exposed to indomethacin 1 mM for 24 h. Relative expression levels that were normalized using the control value from each individual replicate. *: *p* < 0.05; compared with untreated control, calculated using one-way ANOVA and Dunnet’s post-test. (**E**) Cell viability of H1299 cells transfected with the nucleolin-CFP vector and exposed to indomethacin 1 mM for 96 h. ***: *p* < 0.001 and ****: *p* < 0.0001, compared with the control at the same indomethacin concentration, calculated using two-way ANOVA. Data summarize the results of three independent experiments. Original Images can be found in Figure S1.

To investigate the causal relationship between the effects of indomethacin, the increase in SSAT-1 levels, and the levels of nucleolin, we conducted an overexpression experiment of the nucleolin protein in H1299 cells using a nucleolin-CFP (cyan fluorescent protein) system. The overexpression of the protein was confirmed through immunoblotting, where the nucleolin antibody identified two bands: one corresponding to the native nucleolin and the other, with a higher molecular weight, corresponding to the nucleolin tagged with CFP (Figure 4A). Additionally, fluorescence microscopy was performed to visualize the fluorescence emitted by the CFP tag in the transfected cells (Figure 4B). Interestingly, the cells overexpressing the nucleolin-CFP protein did not exhibit an increase in SSAT-1 levels upon indomethacin treatment (Figure 4C,D). Moreover, the impact of indomethacin on cell viability was partially but significantly reversed in the cells overexpressing the nucleolin-CFP protein (Figure 4E).

### 3.5. Indomethacin Has a Synergistic Effect on Cell Viability When Combined with a Selective Polyamine Oxidase Inhibitor

The acetylated polyamines generated by SSAT-1 could be reconverted to non-acetylated polyamines by the activity of the polyamine oxidase enzyme (PAOX) [37]. We previously reported a slight synergistic effect on cell viability between indomethacin and MDL72527, a non-specific inhibitor for PAOX and the spermine oxidase enzyme (SMOX), with a low affinity for PAOX [19]. Recently, methoctramine was described as a potent inhibitor of PAOX, 120-fold more selective for PAOX than SMOX [26].

**Figure 5:**
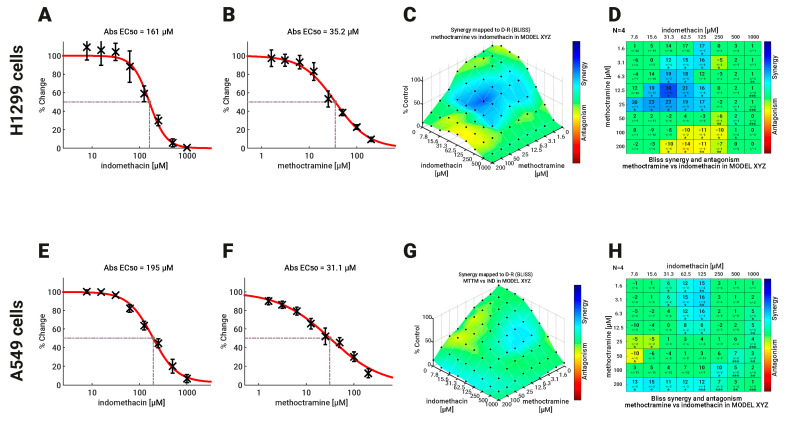
Effect of Indomethacin combined with methoctramine on the viability of NSCLC cells. The effect of the combination of indomethacin with methoctramine on cell viability was measured by resazurin reduction assay. H1299 and A549 cells were exposed to the different drugs for 96 h. For drug combinations, two-fold dilution series, comprising eight concentrations, were mixed in every possible combination. Effects matrices were plotted using COMBENEFIT software, which builds an XYZ model of the combination, using the effect of each drug alone and the Bliss model of drug additivity. (**A**,**B**) Concentration-response curves for indomethacin and methoctramine in H1299 cells. (**C**,**D**) Heatmaps in 3D (**C**) and the statistical analysis (**D**) showing the synergistic effect between indomethacin and methoctramine in H1299 cells. (**E**,**F**) Concentration-response curves for indomethacin and methoctramine in H1299 cells. (**G**,**H**) Heatmaps in 3D (**G**) and the statistical analysis (**H**) showing the synergistic effect between indomethacin and methoctramine in H1299 cells. Differences between the theoretical combinations and empirical data are represented by a number generated for every combination point. Positive numbers represent synergistic combinations, while negative numbers indicate antagonistic interactions. The color code shows combination points that are significantly different from the theoretical model, calculated by the *t*-test. * *p* < 0.05; ** *p* < 0.01 and *** *p* < 0.001. The graphs show the mean ± standard deviation of four independent experiments.

Thus, to further address whether selective PAOX inhibition potentiates the SSAT-1 increase, we evaluated the combined effect of eight concentrations of indomethacin and methoctramine over the viability of H1299 and A549 cells after 96 h of exposure to both drugs at eight concentration dilutions as well as combinations between them. The results are shown in Figure 5. There was no difference between H1299 and A549 cells in the sensitivity to indomethacin and methoctramine, with methoctramine being more potent in both types of lung cancer cells (Figure 5A,B,E,F). The combination was analyzed using Bliss independence model because it better describes the combination of drugs with different modes of action [38]. The combination showed a more potent synergy profile in H1299 cells, with a significant synergy spot when 12.5 µM methoctramine is used together with 31.3 µM indomethacin (Figure 5C,D). In this combination point, a significant increase of 30% in cell death is found compared to the predicted additive model. A slight antagonistic effect (14% of cell viability over the predicted effect) was also detected when 200 µM methoctramine was used (Figure 5D). On the other hand, two zones of slight synergy were found in A549 cells. The first one was shown with methoctramine in the low concentration range (1.6 to 12.5 µM) and indomethacin middle range (31.3 to 125 µM) with a cell death near to 15% over the predicted model. The second synergy zone was observed when methoctramine was used at 200 µM (12 to 15% of lethality increased). A slight spot of antagonism (1% of cell viability over the predicted Bliss model) was found with the combination of 50 µM methoctramine with 7.8 µM indomethacin (Figure 5G,H).

## 4. Discussion

Despite the primary mechanism related to the NSAID effect being the inhibition of the COX-1 and COX-2 enzymes [39], other action mechanisms are also described for this family of drugs. For example, aspirin and ibuprofen inhibit transcription factors such as NF-κB and Activator Protein-1 (AP-1), modifying other kinase activities, such as Inhibitor Of Nuclear Factor Kappa B Kinase Subunit Beta (IKKß), Erk, p38, mitogen-activated protein kinase (MAPK), and o Cyclin-dependent kinases (Cdks) [40]. Consequently, all NSAIDs could not share all these action mechanisms. For example, PPAR-γ is activated by indomethacin, ibuprofen, and naproxen, but not for sodium salicylate or aspirin [40]. Furthermore, among these PPAR-γ activators, indomethacin has a greater affinity for PPAR-γ than ibuprofen or naproxen [15]. On the other hand, the SSAT-1 increase induced by aspirin is associated with the NFκB activation in colon cancer cells [16], in contrast to the evidence indicating that aspirin act as an NFκB inhibitor [40]. Of note, aspirin can also inhibit CDK1 by acetylation [41]. Thus, it cannot be ruled out that the induction of SSAT-1 by aspirin is also related to CDK1 inhibition.

Other NSAIDs also have PPAR-γ-related antitumor activity. Sulindac, an NSAID that induces SSAT-1 in colon cancer cells via PPAR-γ [18], requires this PPAR-γ activity for its antitumor activity in colon cancer models [42]. Celecoxib, a selective COX-2 inhibitor, exerts its ant-inflammatory effect in a PPAR-γ-dependent way in vivo [43]. GW9662 (a PPAR-γ inhibitor) also reverts the apoptotic effect of celecoxib in lung cancer cells [44]. 

Nucleolin is a highly-conserved phosphoprotein located in the nucleolus, and it is involved in rRNA biogenesis, apoptosis regulation, intestinal cell differentiation, and the remodeling of nucleosomes [33]. Downregulation or inhibition of nucleolin yields cell cycle arrest of cell death in several cancer models [45,46,47]. However, the turnover mechanisms are unknown. To date, nucleolin has been said to exert self-cleavage [48] and that this self-cleavage activity is reduced in proliferating cells [49]. However, the mechanisms behind the induction of nucleolin cleavage remain to be solved. 

**Figure 6:**
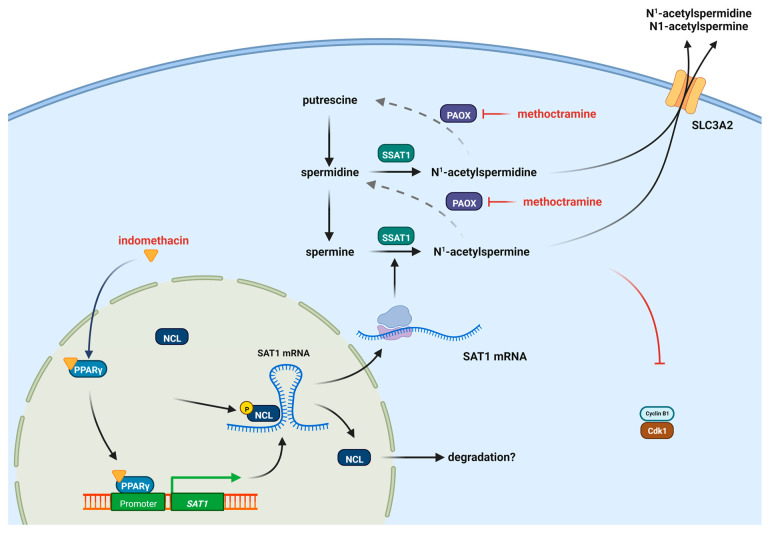
Proposed model of the induction of SSAT-1 by indomethacin and the synergistic effect with methoctramine. Indomethacin can induce the PPAR-γ receptor but also decrease the levels of CDK1, allowing the increase in SSAT-1 synthesis. Methoctramine also inhibits the polyamine oxidase (PAOX) enzyme, increasing the effect of SSAT-1 and having a synergistic effect with indomethacin. The increased acetylated polyamines can be further exported from the cell by the solute carrier 3 family member 2 (SLC3A2) transporter [56]. Figure designed with Biorender.

Nucleolin has been proposed as a target for radio- or chemotherapy due to its role in replication stress induced by DNA damage [50]. Nucleolin is differentially phosphorylated during the cell cycle. During mitosis, threonine phosphorylation is achieved by CDK1, whereas serine phosphorylation by CK2 is extensive during the interphase [51]. In lung cancer cells (A549) phosphorylation of nucleolin in T76 is pivotal for proliferation and migration [52]. Thus, a reduction of CDK1 by indomethacin could lead to a lack of nucleolin phosphorylation at Thr-641/707, inducing nucleolin degradation and allowing the translation of the SSAT-1 mRNA (Figure 6).

The CDK1/CDK2 inhibitors puruvanol A and roscovitine induce cell death in colon cancer cells and increase SSAT-1 levels. Moreover, the blockade of SSAT-1 by using an anti-SSAT-1 siRNA abolishes the apoptotic effect of roscovitine [53]. Puruvanol A also increases the SSAT-1 and PAOX levels in breast cancer cells [54]. However, the induction of SSAT-1 by this CDK1/CDK2 inhibitor seems to also be dependent on the PPAR-γ activity [55]. Thus, it cannot be ruled out that transcriptional and translational mechanisms can cooperate to increase the SSAT-1 levels by indomethacin. 

Figure 6 shows a proposed model where indomethacin has a dual activity, activating PPAR-γ and decreasing nucleolin levels, and generating a transcriptional and translational increase in SSAT-1. This activity could be enhanced by inhibiting the PAOX enzyme, yielding a synergistic effect against lung cancer cells.

## Data Availability

The data presented in this study are available on request from the corresponding author.

## Supplementary Materials

The following supporting information can be downloaded at: https://www.mdpi.com/article/10.3390/biom13091383/s1, Supplementary Figure S1: Full scan (uncropped) immunoblots for figures 1, 2, 3 and 4. Original Images can be found in Figure S1.

## References

[1] Gunaydin C., Bilge S.S. (2018). Effects of Nonsteroidal Anti-Inflammatory Drugs at the Molecular Level. Eurasian J. Med..

[2] Zhang Z., Chen F.L., Shang L.J. (2018). Advances in antitumor effects of NSAIDs. Cancer Manag. Res..

[3] Umezawa S., Higurashi T., Komiya Y., Arimoto J., Horita N., Kaneko T., Iwasaki M., Nakagama H., Nakajima A. (2019). Chemoprevention of colorectal cancer: Past, present, and future. Cancer Sci..

[4] Zhao X.P., Xu Z., Li H.S. (2017). NSAIDs Use and Reduced Metastasis in Cancer Patients: Results from a meta-analysis. Sci. Rep..

[5] Shaji S., Smith C., Forget P. (2021). Perioperative NSAIDs and Long-Term Outcomes after cancer Surgery: A Systematic Review and Meta-analysis. Curr. Oncol. Rep..

[6] Lim W.Y., Chuah K.L., Eng P., Leong S.S., Lim E., Lim T.K., Ng A., Poh W.T., Tee A., Teh M. (2012). Aspirin and non-aspirin non-steroidal anti-inflammatory drug use and risk of lung cancer. Lung Cancer.

[7] Olsen J.H., Friis S., Poulsen A.H., Fryzek J., Harving H., Tjonneland A., Sorensen H.T., Blot W. (2008). Use of NSAIDs, smoking and lung cancer risk. Br. J. Cancer.

[8] McCormack V.A., Hung R.J., Brenner D.R., Bickeboller H., Rosenberger A., Muscat J.E., Lazarus P., Tjonneland A., Friis S., Christiani D.C. (2011). Aspirin and NSAID use and lung cancer risk: A pooled analysis in the International Lung Cancer Consortium (ILCCO). Cancer Causes Control..

[9] Jara-Gutierrez A., Baladron V. (2021). The Role of Prostaglandins in Different Types of Cancer. Cells.

[10] Kazberuk A., Zareba I., Palka J., Surazynski A. (2020). A novel plausible mechanism of NSAIDs-induced apoptosis in cancer cells: The implication of proline oxidase and peroxisome proliferator-activated receptor. Pharmacol. Rep..

[11] Kolawole O.R., Kashfi K. (2022). NSAIDs and Cancer Resolution: New Paradigms beyond Cyclooxygenase. Int. J. Mol. Sci..

[12] Matsunaga S., Asano T., Tsutsuda-Asano A., Fukunaga Y. (2006). Indomethacin overcomes doxorubicin resistance with inhibiting multi-drug resistance protein 1 (MRP1). Cancer Chemother. Pharmacol..

[13] Zhou Y., Wang S., Ying X., Wang Y., Geng P., Deng A., Yu Z. (2017). Doxorubicin-loaded redox-responsive micelles based on dextran and indomethacin for resistant breast cancer. Int. J. Nanomed..

[14] Amanullah A., Mishra R., Upadhyay A., Reddy P.P., Das R., Mishra A. (2018). Indomethacin elicits proteasomal dysfunctions develops apoptosis through mitochondrial abnormalities. J. Cell Physiol..

[15] Jaradat M.S., Wongsud B., Phornchirasilp S., Rangwala S.M., Shams G., Sutton M., Romstedt K.J., Noonan D.J., Feller D.R. (2001). Activation of peroxisome proliferator-activated receptor isoforms and inhibition of prostaglandin H(2) synthases by ibuprofen, naproxen, and indomethacin. Biochem. Pharmacol..

[16] Babbar N., Gerner E.W., Casero R.A. (2006). Induction of spermidine/spermine N1-acetyltransferase (SSAT) by aspirin in Caco-2 colon cancer cells. Biochem. J..

[17] Turchanowa L., Dauletbaev N., Milovic V., Stein J. (2001). Nonsteroidal anti-inflammatory drugs stimulate spermidine/spermine acetyltransferase and deplete polyamine content in colon cancer cells. Eur. J. Clin. Investig..

[18] Babbar N., Ignatenko N.A., Casero R.A., Gerner E.W. (2003). Cyclooxygenase-independent induction of apoptosis by sulindac sulfone is mediated by polyamines in colon cancer. J. Biol. Chem..

[19] López-Contreras F., Muñoz-Uribe M., Pérez-Laines J., Ascencio-Leal L., Rivera-Dictter A., Martin-Martin A., Burgos R.A., Alarcon P., López-Muñoz R. (2020). Searching for Drug Synergy Against Cancer Through Polyamine Metabolism Impairment: Insight Into the Metabolic Effect of Indomethacin on Lung Cancer Cells. Front. Pharmacol..

[20] Pegg A.E. (2008). Spermidine/spermine-N(1)-acetyltransferase: A key metabolic regulator. Am. J. Physiol. Endocrinol. Metab..

[21] Casero R.A., Murray Stewart T., Pegg A.E. (2018). Polyamine metabolism and cancer: Treatments, challenges and opportunities. Nat. Rev. Cancer.

[22] Nowotarski S.L., Woster P.M., Casero R.A. (2013). Polyamines and cancer: Implications for chemotherapy and chemoprevention. Expert Rev. Mol. Med..

[23] Min J.Z., Matsumoto A., Li G., Jiang Y.Z., Yu H.F., Todoroki K., Inoue K., Toyo’oka T. (2014). A quantitative analysis of the polyamine in lung cancer patient fingernails by LC-ESI-MS/MS. Biomed. Chromatogr..

[24] Mandal S., Mandal A., Johansson H.E., Orjalo A.V., Park M.H. (2013). Depletion of cellular polyamines, spermidine and spermine, causes a total arrest in translation and growth in mammalian cells. Proc. Natl. Acad. Sci. USA.

[25] Novita Sari I., Setiawan T., Seock Kim K., Toni Wijaya Y., Won Cho K., Young Kwon H. (2021). Metabolism and function of polyamines in cancer progression. Cancer Lett..

[26] Di Paolo M.L., Cervelli M., Mariottini P., Leonetti A., Polticelli F., Rosini M., Milelli A., Basagni F., Venerando R., Agostinelli E. (2019). Exploring the activity of polyamine analogues on polyamine and spermine oxidase: Methoctramine, a potent and selective inhibitor of polyamine oxidase. J. Enzyme Inhib. Med. Chem..

[27] Martinez M.E., O’Brien T.G., Fultz K.E., Babbar N., Yerushalmi H., Qu N., Guo Y.J., Boorman D., Einspahr J., Alberts D.S. (2003). Pronounced reduction in adenoma recurrence associated with aspirin use and a polymorphism in the ornithine decarboxylase gene. Proc. Natl. Acad. Sci. USA.

[28] Mitsudomi T., Steinberg S.M., Nau M.M., Carbone D., D’Amico D., Bodner S., Oie H.K., Linnoila R.I., Mulshine J.L., Minna J.D. (1992). p53 gene mutations in non-small-cell lung cancer cell lines and their correlation with the presence of ras mutations and clinical features. Oncogene.

[29] Lehman T.A., Bennett W.P., Metcalf R.A., Welsh J.A., Ecker J., Modali R.V., Ullrich S., Romano J.W., Appella E., Testa J.R. (1991). p53 mutations, ras mutations, and p53-heat shock 70 protein complexes in human lung carcinoma cell lines. Cancer Res..

[30] Foucquier J., Guedj M. (2015). Analysis of drug combinations: Current methodological landscape. Pharmacol. Res. Perspect..

[31] Di Veroli G.Y., Fornari C., Wang D., Mollard S., Bramhall J.L., Richards F.M., Jodrell D.I. (2016). Combenefit: An interactive platform for the analysis and visualization of drug combinations. Bioinformatics.

[32] Perez-Leal O., Barrero C.A., Clarkson A.B., Casero R.A., Merali S. (2012). Polyamine-Regulated Translation of Spermidine/Spermine-N-1-Acetyltransferase. Mol. Cell. Biol..

[33] Tajrishi M.M., Tuteja R., Tuteja N. (2011). Nucleolin: The most abundant multifunctional phosphoprotein of nucleolus. Commun. Integr. Biol..

[34] Zhang X., Xiao S., Rameau R.D., Devany E., Nadeem Z., Caglar E., Ng K., Kleiman F.E., Saxena A. (2018). Nucleolin phosphorylation regulates PARN deadenylase activity during cellular stress response. RNA Biol..

[35] Wang S.-A., Li H.-Y., Hsu T.-I., Chen S.-H., Wu C.-J., Chang W.-C., Hung J.-J. (2011). Heat Shock Protein 90 Stabilizes Nucleolin to Increase mRNA Stability in Mitosis. J. Biol. Chem..

[36] Shiff S.J., Koutsos M.I., Qiao L., Rigas B. (1996). Nonsteroidal antiinflammatory drugs inhibit the proliferation of colon adenocarcinoma cells: Effects on cell cycle and apoptosis. Exp. Cell Res..

[37] Seiler N. (2004). Catabolism of polyamines. Amino Acids.

[38] Fitzgerald J.B., Schoeberl B., Nielsen U.B., Sorger P.K. (2006). Systems biology and combination therapy in the quest for clinical efficacy. Nat. Chem. Biol..

[39] Mazaleuskaya L.L., Ricciotti E. (2020). Druggable Prostanoid Pathway. Adv. Exp. Med. Biol..

[40] Tegeder I., Pfeilschifter J., Geisslinger G. (2001). Cyclooxygenase-independent actions of cyclooxygenase inhibitors. FASEB J..

[41] Dachineni R., Kumar D.R., Calegari E., Kesharwani S.S., Sankaranarayanan R., Seefeldt T., Tumala H., Bhat G.J. (2017). Salicylic acid metabolites and derivatives inhibit CDK activity: Novel insights into aspirin’s chemopreventive effects against colorectal cancer. Int. J. Oncol..

[42] Nikitakis N.G., Hebert C., Lopes M.A., Reynolds M.A., Sauk J.J. (2002). PPAR gamma-mediated antineoplastic effect of NSAID sulindac on human oral squamous carcinoma cells. Int. J. Cancer.

[43] Houshmand G., Naghizadeh B., Ghorbanzadeh B., Ghafouri Z., Goudarzi M., Mansouri M.T. (2020). Celecoxib inhibits acute edema and inflammatory biomarkers through peroxisome proliferator-activated receptor-gamma in rats. Iran. J. Basic Med. Sci..

[44] Ramer R., Walther U., Borchert P., Laufer S., Linnebacher M., Hinz B. (2013). Induction but not inhibition of COX-2 confers human lung cancer cell apoptosis by celecoxib. J. Lipid Res..

[45] Wu C.D., Chou H.W., Kuo Y.S., Lu R.M., Hwang Y.C., Wu H.C., Lin C.T. (2012). Nucleolin antisense oligodeoxynucleotides induce apoptosis and may be used as a potential drug for nasopharyngeal carcinoma therapy. Oncol. Rep..

[46] Ugrinova I., Monier K., Ivaldi C., Thiry M., Storck S., Mongelard F., Bouvet P. (2007). Inactivation of nucleolin leads to nucleolar disruption, cell cycle arrest and defects in centrosome duplication. BMC Mol. Biol..

[47] Jain N., Zhu H., Khashab T., Ye Q., George B., Mathur R., Singh R.K., Berkova Z., Wise J.F., Braun F.K. (2018). Targeting nucleolin for better survival in diffuse large B-cell lymphoma. Leukemia.

[48] Fang S.H., Yeh N.H. (1993). The self-cleaving activity of nucleolin determines its molecular dynamics in relation to cell proliferation. Exp. Cell Res..

[49] Chen C.M., Chiang S.Y., Yeh N.H. (1991). Increased stability of nucleolin in proliferating cells by inhibition of its self-cleaving activity. J. Biol. Chem..

[50] Kawamura K., Qi F., Meng Q., Hayashi I., Kobayashi J. (2019). Nucleolar protein nucleolin functions in replication stress–induced DNA damage responses. J. Radiat. Res..

[51] Belenguer P., Caizergues-Ferrer M., Labbé J.C., Dorée M., Amalric F. (1990). Mitosis-specific phosphorylation of nucleolin by p34cdc2 protein kinase. Mol. Cell. Biol..

[52] Huang F.F., Wu Y.Y., Tan H., Guo T.Y., Zhang K., Li D.Q., Tong Z.Y. (2019). Phosphorylation of nucleolin is indispensable to its involvement in the proliferation and migration of non-small cell lung cancer cells. Oncol. Rep..

[53] Coker A., Arisan E.D., Palavan-Unsal N. (2012). Silencing of the polyamine catabolic key enzyme SSAT prevents CDK inhibitor-induced apoptosis in Caco-2 colon cancer cells. Mol. Med. Rep..

[54] Obakan P., Arisan E.D., Ozfiliz P., Coker-Gurkan A., Palavan-Unsal N. (2014). Purvalanol A is a strong apoptotic inducer via activating polyamine catabolic pathway in MCF-7 estrogen receptor positive breast cancer cells. Mol. Biol. Rep..

[55] Obakan P., Yildirim S., Ozturk M.B., Berrak O., Gurkan A.C., Arisan E.D., Unsal Z.N. (2015). CDK inhibitors-induced SSAT expression requires NF kappa B and PPAR gamma in MCF-7 breast cancer cells. Turk. J. Biol..

[56] Affronti H.C., Rowsam A.M., Pellerite A.J., Rosario S.R., Long M.D., Jacobi J.J., Bianchi-Smiraglia A., Boerlin C.S., Gillard B.M., Karasik E. (2020). Pharmacological polyamine catabolism upregulation with methionine salvage pathway inhibition as an effective prostate cancer therapy. Nat. Commun..

