# Supplementary material for: Indomethacin Induces Spermidine/Spermine-N1-Acetyltransferase-1 via the Nucleolin-CDK1 Axis and Synergizes with the Polyamine Oxidase Inhibitor Methoctramine in Lung Cancer Cells
Source: Biomolecules. 2023 Sep 12;13(9):1383. doi: 10.3390/biom13091383 (PMC10526249; doi:10.3390/biom13091383)
Supplement: Supplementary file 1 [file biomolecules-13-01383-s001.zip › biomolecules-2515886-supplementary.pdf]

biomolecules

MDPI

Supplementary material

Indomethacin induces spermidine/spermine-N[1]-acetyltransferase-1 via the nucleolin-CDK1 axis and synergizes with the polyamine oxidase inhibitor methoctramine in lung cancer cells.

Neudo Buelvas[1], Isidora Ugarte-Vio[1], Laura Asencio-Leal[1], Matías Muñoz-Uribe[1], Antonia Martin-Martin[1], Alejandro Rojas-Fernández[2], José A. Jara[3], Julio Tapia[4], María Elena Arias[5], Rodrigo A. López-Muñoz[1]*

[1] Instituto de Farmacología y Morfofisiología, Facultad de Ciencias Veterinarias, Universidad Austral de Chile, Valdivia, Chile
[2] Instituto de Medicina, Facultad de Medicina, Universidad Austral de Chile Valdivia, Chile
[3] Instituto de Investigaciones en Ciencias Odontológicas (ICOD), Facultad de Odontología, Universidad de Chile, Santiago, Chile
[4] Programa de Biología Celular y Molecular, Instituto de Ciencias Biomédicas, Facultad de Medicina, Universidad de Chile, Santiago, Chile
[5] Departamento de Producción Agropecuaria. Universidad de La Frontera. Temuco, Chile
* Correspondence: Rodrigo López-Muñoz; Email: rodrigo.lopez@uach.cl; Tel.: +56-632-444321 (R. L.-M.)

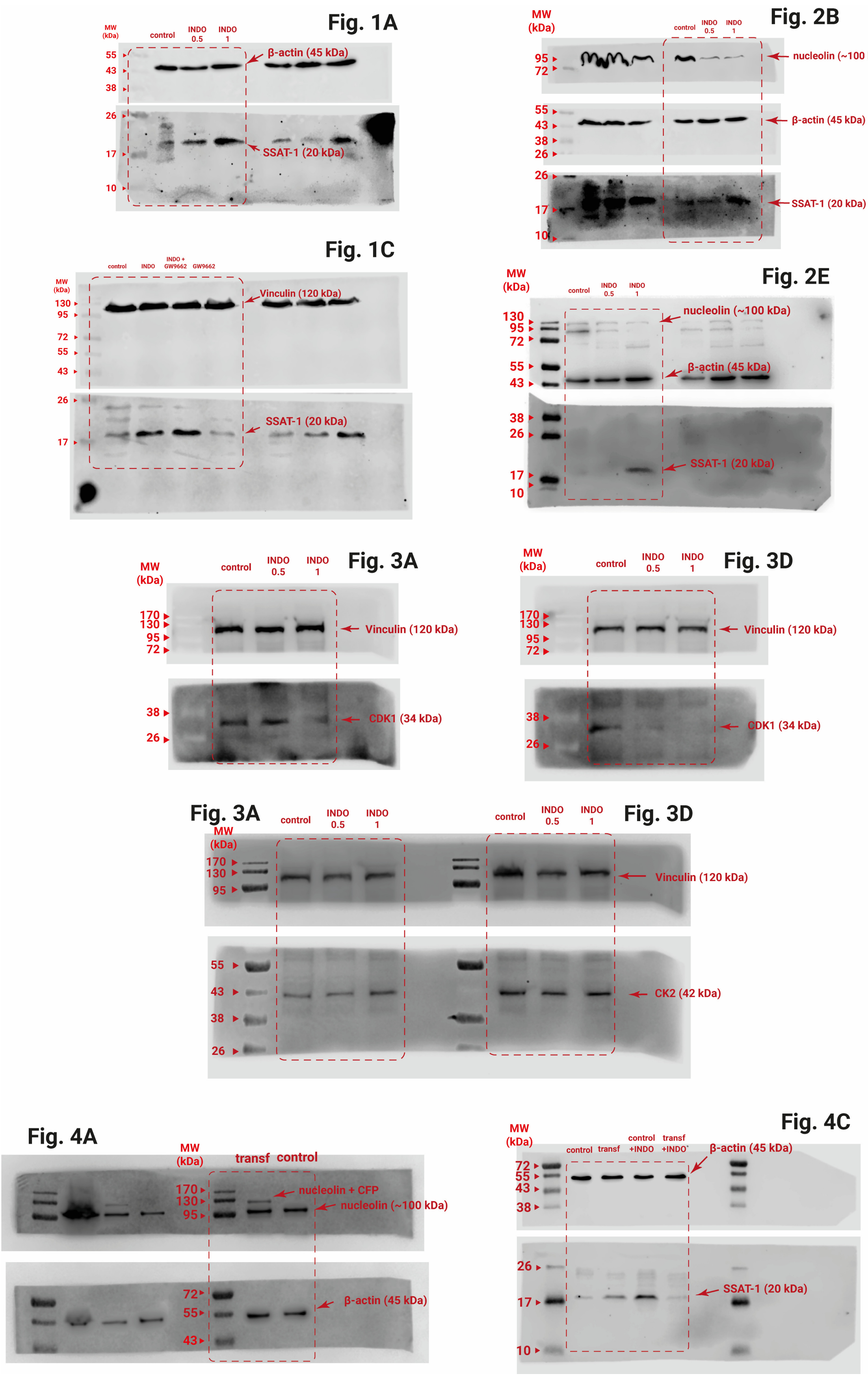

Scheme 1. Full scan (uncropped) immunoblots for figures 1, 2, 3 and 4. Molecular weight markers are indicated with red triangles. The figure subpanel is indicated for each blot. Red dotted squares indicate the selected panels shown in each figure.

© 2023 by the authors. Submitted for possible open access publication under the terms and conditions of the Creative Commons Attribution (CC BY) license (http://creativecommons.org/licenses/by/4.0/).
